# Supplementary material for: Nivolumab after Induction Chemotherapy in Previously Treated Non-Small-Cell Lung Cancer Patients with Low PD-L1 Expression
Source: Cancers (Basel). 2023 Sep 7;15(18):4460. doi: 10.3390/cancers15184460 (PMC10526182; doi:10.3390/cancers15184460)
Supplement: Supplementary file 1 [file cancers-15-04460-s001.zip › Supplementary Table S1.pdf]

Supplementary Table S1. Gene set enrichment analysis (GSEA) was applied to identify pathways using the KEGG gene set of DAVID KEGG pathway analysis (Pvalue < 0.05)

Test	Term	Count	PValue	FDR	Genes
pre	Complement and coagulation cascades	17	8.79E-12	2.06E-09	CFD, CR2, CFH, F10, SERPINC1, CFI, SERPINE1, F13A1, TFPI, CLU, F5, C2, F8, C6, CFHR2, CFHR4, F13B
	Cholesterol metabolism	9	7.14E-06	0.0008	CETP, LIPC, SORT1, ANGPTL8, ANGPTL3, LPL, APOA1, APOE, APOB
	ECM-receptor interaction	9	0.0004	0.030	RELN, TNXB, ITGA2, COL6A2, COL6A1, CHAD, LAMB1, ITGA5, THBS1
	Phagosome	11	0.001	0.058	COLEC11, LAMP1, TFRC, ITGA2, MRC1, HLA-A, ITGA5, THBS1, CORO1A, CTSS, HLA-DRB1
	Biosynthesis of amino acids	7	0.004	0.185	PKM, GOT1, PGAM1, IDH1, ASL, MTR, ENO1
	Protein digestion and absorption	8	0.005	0.185	COL15A1, PRSS1, CPA1, CPB1, XPNPEP2, COL6A2, COL6A1, PRCP
	Focal adhesion	11	0.007	0.250	CDC42, RELN, TNXB, ITGA2, COL6A2, COL6A1, CHAD, LAMB1, ITGA5, THBS1, MYL9
	Shigellosis	12	0.011	0.332	ACTR3, CDC42, CAST, ACTR2, ARPC2, ARPC3, CCL5, UBE2N, ITGA5, MAPK14, MYL9, SEPTIN7
	Hematopoietic cell lineage	7	0.015	0.387	CR2, TFRC, ITGA2, IL1R2, ITGA5, CD33, HLA-DRB1
	Bacterial invasion of epithelial cells	6	0.020	0.456	ACTR3, CDC42, ACTR2, ARPC2, ARPC3, ITGA5
	Glutathione metabolism	5	0.028	0.561	GCLC, G6PD, GGT7, IDH1, PRDX6
	Carbon metabolism	7	0.029	0.561	G6PD, PKM, GOT1, PGAM1, IDH1, ENO1, ACAT2
	Staphylococcus aureus infection	6	0.045	0.772	CFD, CFH, CFI, DSG1, HLA-DRB1, C2
	Glycolysis / Gluconeogenesis	5	0.046	0.772	LDHA, PKM, PGAM1, PGM2, ENO1
FC	Complement and coagulation cascades	20	3.42E-15	8.00E-13	C1QB, CFD, C1QA, SERPIND1, C1R, F13A1, C8B, CLU, KNG1, F5, C2, SERPINA5, VTN, F8, C6, CFHR2, CFHR5, F13B, C1QC, MBL2
	Cholesterol metabolism	15	8.02E-13	9.38E-11	CETP, LRP1, APOA2, APOA1, APOC3, LCAT, APOA4, LIPC, APOC2, APOC1, ANGPTL3, APOE, APOB, LDLR, PLTP
	Staphylococcus aureus infection	13	4.78E-07	3.73E-05	C1QB, CFD, C1QA, C1R, DEFA1, C2, FCGR3A, HLA-DPB1, DSG1, DEFA1B, CAMP, C1QC, MBL2
	Phagosome	15	2.28E-06	0.0001	ITGB1, COLEC11, TFRC, C1R, ITGA2, SFTPD, HLA-A, THBS1, COMP, FCGR3A, TUBB1, LAMP2, HLA-DPB1, ITGA5, MBL2
	ECM-receptor interaction	11	1.06E-05	0.0005	ITGB1, COMP, VTN, RELN, LAMB2, ITGA2, TNC, COL6A3, ITGA5, THBS1, GP5
	Focal adhesion	16	1.33E-05	0.001	PDGFRB, ITGB1, LAMB2, SRC, ITGA2, TNC, IGF1, THBS1, CRKL, COMP, VTN, RELN, CAPN2, COL6A3, MAPK1, ITGA5
	Pertussis	8	0.001	0.028	ITGB1, C1QB, C1QA, C1R, MAPK1, ITGA5, C1QC, C2
	Coronavirus disease - COVID-19	13	0.003	0.071	C1QB, CFD, C1QA, C1R, MMP3, F13A1, C8B, C2, C6, MAPK1, F13B, C1QC, MBL2
	TGF-beta signaling pathway	8	0.003	0.071	TGFB1, MAPK1, THBS1, NEO1, LTBP1, NBL1, INHBC, INHBE
	Proteoglycans in cancer	12	0.003	0.071	ITGB1, VTN, TGFB1, SRC, LUM, ITGA2, IGF2, MAPK1, IGF1, ITGA5, THBS1, MMP9
	Chagas disease	8	0.005	0.099	C1QB, C1QA, TGFB1, CCL5, GNAQ, MAPK1, KNG1, C1QC
	PI3K-Akt signaling pathway	16	0.006	0.108	PDGFRB, ITGB1, LAMB2, ITGA2, TNC, IGF2, IGF1, OSMR, THBS1, HSP90B1, COMP, VTN, RELN, COL6A3, MAPK1, ITGA5
	Systemic lupus erythematosus	9	0.006	0.116	C1QB, C1QA, FCGR3A, C6, C1R, HLA-DPB1, C8B, C1QC, C2
	Hypertrophic cardiomyopathy	7	0.010	0.165	ITGB1, TGFB1, ITGA2, LMNA, IGF1, ITGA5, TTN
	Lipid and atherosclerosis	11	0.012	0.173	SRC, CCL5, MMP3, MAPK1, APOA1, APOA4, APOB, LDLR, MMP9, ATF6, HSP90B1
	Platelet activation	8	0.013	0.173	ITGB1, COL3A1, SRC, GNAQ, ITGA2, MAPK1, GP5, FERMT3
	Dilated cardiomyopathy	7	0.013	0.173	ITGB1, TGFB1, ITGA2, LMNA, IGF1, ITGA5, TTN
	Regulation of actin cytoskeleton	11	0.013	0.173	PDGFRB, ITGB1, ACTR2, GSN, SRC, ARPC3, ITGA2, MAPK1, ITGA5, KNG1, CRKL
	PPAR signaling pathway	6	0.018	0.222	FABP5, APOA2, ME1, APOC3, APOA1, PLTP
	Bacterial invasion of epithelial cells	6	0.020	0.234	ITGB1, ACTR2, SRC, ARPC3, ITGA5, CRKL
	Yersinia infection	8	0.021	0.239	ITGB1, ACTR2, SRC, ARPC3, GNAQ, MAPK1, ITGA5, CRKL
	Human papillomavirus infection	13	0.037	0.395	PDGFRB, ITGB1, LAMB2, ITGA2, TNC, HLA-A, THBS1, COMP, VTN, RELN, COL6A3, MAPK1, ITGA5
	Salivary secretion	6	0.039	0.398	CST3, CST1, GNAQ, ATP1B1, LYZ, CAMP
	Fc gamma R-mediated phagocytosis	6	0.047	0.434	ACTR2, FCGR3A, GSN, ARPC3, MAPK1, CRKL

	Prostate cancer	6	0.047	0.434	PDGFRB, MMP3, MAPK1, IGF1, MMP9, HSP90B1
post	Complement and coagulation cascades	13	3.16E-08	6.61E-06	C1QB, FGA, C1QA, CLU, KNG1, SERPINA5, F7, VTN, F8, CFHR2, C7, CFHR4, VSIG4
	Focal adhesion	17	4.89E-07	5.11E-05	PDGFRB, TNXB, SRC, ITGA2B, IGF1, THBS4, CRKL, MYLK, COMP, VTN, COL6A2, COL6A1, CHAD, KDR, CAPN2, ITGA6, TLN1
	Cholesterol metabolism	9	2.77E-06	0.0002	CETP, APOH, ANGPTL8, APOA2, ANGPTL3, APOC3, LCAT, APOA1, APOA4
	Phagosome	13	1.54E-05	0.0008	HLA-A, TUBB4B, MPO, TUBA4A, CTSS, THBS4, COMP, MARCO, TUBA1A, TUBB1, MRC1, LAMP2, RAB7A
	ECM-receptor interaction	10	2.42E-05	0.001	COMP, VTN, TNXB, COL6A2, ITGA2B, COL6A1, CHAD, ITGA6, NPNT, THBS4
	Regulation of actin cytoskeleton	14	0.0002	0.007	PDGFRB, ACTR3, ACTR2, GSN, SRC, ITGA2B, KNG1, CRKL, MYLK, C7, ARPC3, MYH9, PIP4K2A, ITGA6
	Bacterial invasion of epithelial cells	7	0.002	0.072	ACTR3, ACTR2, SRC, ARPC3, SEPTIN2, HCLS1, CRKL
	Pathogenic Escherichia coli infection	10	0.008	0.185	ACTR3, ACTR2, TUBA1A, SRC, ARPC3, TUBB1, MYH9, HCLS1, TUBB4B, TUBA4A
	Lysosome	8	0.009	0.185	NAGLU, LAMP2, HEXA, CTSF, GUSB, CTSD, GLA, CTSS
	Protein digestion and absorption	7	0.010	0.185	COL3A1, CPA1, CPB1, XPNPEP2, COL6A2, COL6A1, PRCP
	PI3K-Akt signaling pathway	14	0.010	0.185	PDGFRB, CSF1R, TNXB, ITGA2B, IGF1, OSMR, THBS4, COMP, VTN, COL6A2, COL6A1, CHAD, KDR, ITGA6
	Apoptosis	8	0.011	0.185	TUBA1A, CAPN2, CTSF, SPTAN1, CTSD, CTSS, TUBA4A, LMNB1
	Gap junction	6	0.020	0.322	PDGFRB, TUBA1A, SRC, TUBB1, TUBB4B, TUBA4A
	Platelet activation	7	0.023	0.344	FGA, COL3A1, SRC, ITGA2B, TLN1, RASGRP2, MYLK
	Tight junction	8	0.031	0.405	ACTR3, ACTR2, TUBA1A, SRC, ARPC3, MYH9, HCLS1, TUBA4A
	Rap1 signaling pathway	9	0.034	0.405	PDGFRB, CSF1R, SRC, ITGA2B, KDR, IGF1, TLN1, RASGRP2, CRKL
	Human papillomavirus infection	12	0.034	0.405	PDGFRB, COMP, VTN, PKM, TNXB, COL6A2, ITGA2B, COL6A1, CHAD, ITGA6, HLA-A, THBS4
	Endocytosis	10	0.035	0.405	ACTR3, EHD1, ACTR2, CAPZB, SRC, ARPC3, CAPZA1, CAPZA2, HLA-A, RAB7A
	Glycosaminoglycan degradation	3	0.044	0.485	NAGLU, HEXA, GUSB
