# Supplementary material for: Nivolumab after Induction Chemotherapy in Previously Treated Non-Small-Cell Lung Cancer Patients with Low PD-L1 Expression
Source: Cancers (Basel). 2023 Sep 7;15(18):4460. doi: 10.3390/cancers15184460 (PMC10526182; doi:10.3390/cancers15184460)
Supplement: Supplementary file 1 [file cancers-15-04460-s001.zip › Supplementary Table S2.pdf]

Supplementary Table S2. Clinicopathologic features of independent cohort

	**N =167**
**Sex, *n* (%)**	
Male	112 (67)
Female	55 (33)
**Age, median (range)**	63 (33-81)
**Age group, *n* (%)**	
<65 y	94 (56)
≥65 y	73 (44)
**Smoking, *n* (%)**	
Never	50 (30)
Former	96 (57)
Current	21 (13)
**Pack-year, mean (95% CI)**	26.7 (22.9-30.6)
**Histology, *n* (%)**	
Adenocarcinoma	123 (75)
Squamous carcinoma	38 (23)
Others	6 (3)
**ECOG PS group, *n* (%)**	
0-1	127 (76)
2-3	40 (24)
**Line, *n* (%)**	
≤2	79 (47)
≥3	88 (53)
**Regimen, *n* (%)**	
Atezolizumab	67 (40)
Nivolumab	53 (32)
Pembrolizumab	47 (28)
**PDL1 expression, *n* (%)**	
<10%	70 (42)
≥10%	94 (56)
unknown	3 (2)
**TFRC level (*μ*g/mL), mean (95% CI)**	3.65 (3.19-4.11)
**TFRC group, total *n* =50 (%)**	
Low	25 (50)
High	25 (50)

ECOG PS, Eastern Cooperative Oncology Group performance status
